# Cytotoxicity of subcritical water extracts obtained from byproducts generated at commercial pecan shelling operations on cancer cells

**DOI:** 10.1186/s40643-023-00666-z

**Published:** 2023-07-28

**Authors:** Canan Sevimli Gur, Nurhan Turgut Dunford, Zinar Pinar Gumus

**Affiliations:** 1 Department of Basic Pharmaceutical Sciences, Katip Celebi University, Izmir, Turkey; 2 Department of Biosystems and Agricultural Engineering and Robert M. Kerr Food and Agricultural Products Center, Oklahoma State University, FAPC Room 103, Stillwater, OK 74078-6055 USA; 3 Central Research Test and Analysis Laboratory Application and Research Center (EGE-MATAL), Ege University, Bornova, İzmir Turkey

**Keywords:** Subcritical water extracts, Pecan shell extracts, Cancer cell lines, Pecan variety

## Abstract

This study examined potential of the extracts obtained from the byproducts generated at commercial pecan nut-shelling operations in cancer treatment. The subcritical water extracts obtained from two varieties, Native and Pawnee, were analyzed for their phenolic contents and compositions. Effects of the extracts on viability and IC50 of the human cell lines representing a broad range of cancer types, cervical, lung, skin, breast, colon and prostate cancers, were investigated. Although the effect of the temperature on the phenolic contents and compositions of the extracts was not statistically significant, the influence of the variety was extensive. The pecan shell extracts were not cytotoxic to the healthy cell line Vero in the concentration range examined. Some of the pecan shell extracts had greater efficay than Doxorubicin, a drug used in cancer chemotherapy, in reducing cancer cell viability. This study is novel and practical implications of the data generated in this study are noteworthy, because this is the first report on the beneficial effects of subcritical water extracts obtained from pecan shelling industry byproducts on a broad range of cancer cell lines. It is likely that the experimental data presented in this study will support and encourage future research on the biological pathways involved in the interactions of the cancer cells and the extracts. The findings of this study will facilitate research on downstream processing and purification of the crude extracts exhibiting high cancer cell cytotoxcity, potentially improving the final product efficacy and lead to commercial applications.

**Graphical Abstract:**

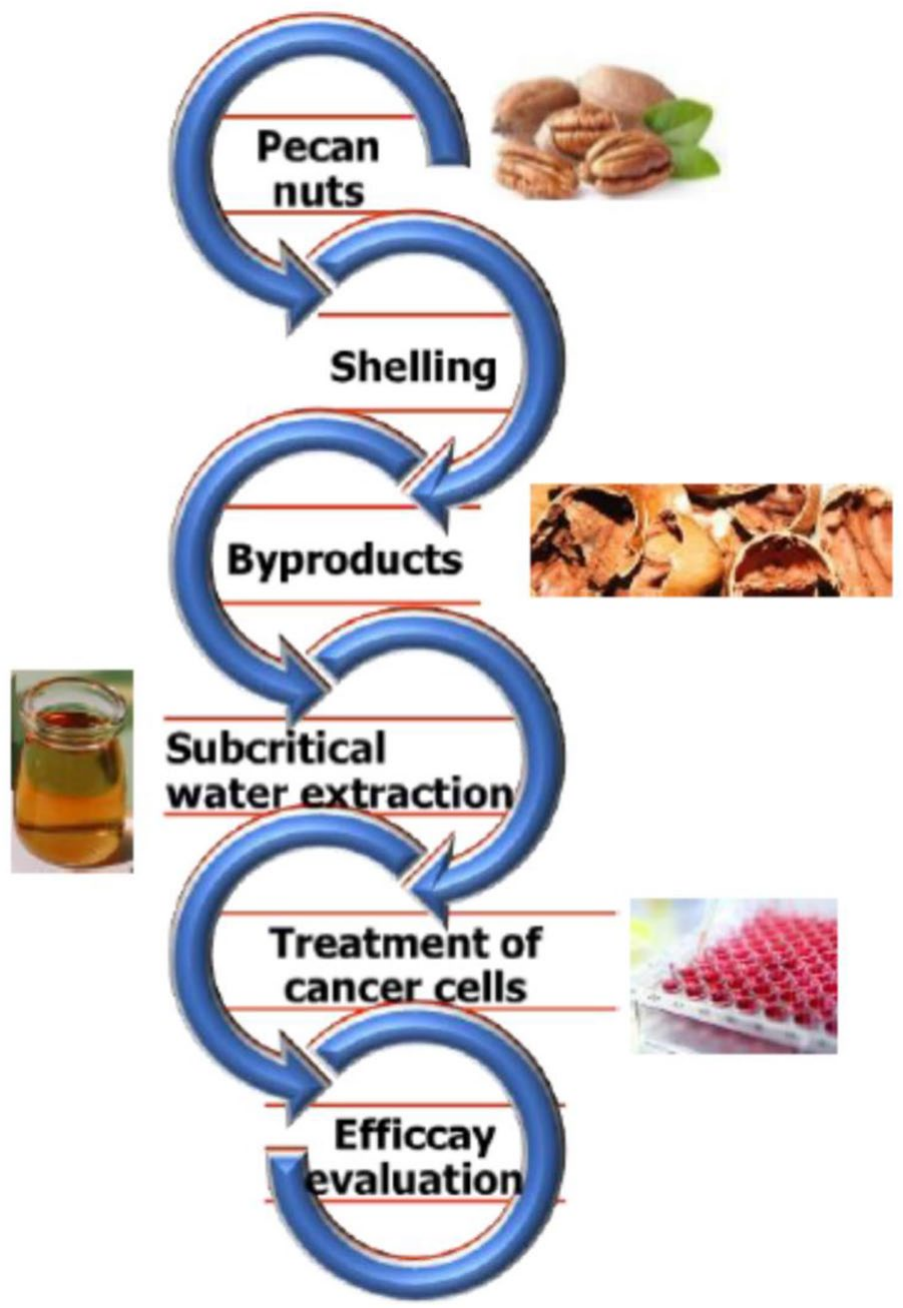

## Introduction

Pecan (*Carya illinoinensis*) trees are native to the United States and grown in all the southern states, including Oklahoma. Various health benefits of tree nuts like pecans have been reported (Cogan et al. 2023; Guarneiri et al. 2021; Tong et al. 2022). The US Food and Drug Administration (FDA) and Dietary guidelines emphasize the beneficial health effects of nut consumption (FDA 2003, 2015). The latter effect is attributed to the high concentration of proteins and other phytochemicals naturally present in nut meat.

Like other tree nuts, the edible part of pecan nut, also referred to as meat or kernel, is enclosed in a hard shell. Whole nuts are shelled to separate hard shell from the nut meat (Sims 1994). Hence, pecan nutshells are byproducts of the shelling industry and usually marketed as gardening aid (mulch), a low value product. About 40–50% of the whole nut weight is comprised of shells. Considering that United States produces 80–90% of the world’s pecan nut production and over 300 million pounds (3.95 million tons in 2021) of pecan nuts are produces in the US, about 2 million tons of pecan shells would be available for processing annually. Thus, shells have economic significance to pecan processors. Finding high value applications for pecan nutshells would benefit not only the processors but also other stake holders including growers and communities who rely on this industry.

Presence of phenolic compounds and flavonoids in pecan shells is well known (Atanasov et al. 2018; do Prado et al. 2014; Prado et al. 2009; Trevizol et al. 2011). Pecan shells contain higher concentrations of phenols and tannins than that found in the edible part of the nuts. The latter compounds are potent antioxidants providing nutritional and medicinal benefits (do Prado et al. 2009). Antioxidants quench oxidation reactions that can damage the body at the cellular level leading to increased risk of many diseases. Previous studies examined the antioxidant capacity of pecan shell extracts obtained using various types of solvents, extraction methods and pecan varieties (Cason et al. 2021; Dunford et al. 2022; Prado et al. 2009; Sevimli-Gur et al. 2021). This study investigates the effect of pecan shell extracts on cancer cells.

Cancer is characterized as a proliferative disorder that causes uncontrolled cell growth. Anticancer activity of plant based polyphenolic compounds has been reported earlier (Baek et al. 2004). A study by Hilbig et al. (2018) have shown that pecan nut shell extracts effectively reduced viability of the breast cancer tumor cells and intensified cell death. Another study by Porto et al. (2016) reported that pecan shell extracts did not induce DNA damage or mutagenicity.

In a previous study, we reported that chemical composition of the pecan shells hand-separated from the nut meat in a laboratory was significantly different than that of shells generated at a commercial pecan nut-shelling operation and majority of the research studies carried out with the pecan shells used hand-separated shells to obtain the extracts (Sevimli-Gur et al. 2021). It is most likely that commercial value-added pecan processing operations would use industrial byproducts rather than hand-separated shells. Furthermore, an extensive study on the effect of pecan shell extracts obtained from different varieties is lacking. It is well established that cultivar type and growth location may have a significant effect on the amount and composition of the phytochemicals in plants (do Prado et al. 2013). Moreover, chemical composition of the extracts is influenced by the extraction method, potentially impacting their biological activity. This study examined subcritical water extraction of pecan shells. The reason for choosing the latter technology is several fold. Water is an environmentally benign solvent that has been used in many applications. However, freshwater scarcity and its polarity are some of the drawbacks limiting its broader use. Subcritical water extraction allows selective extraction via modification of the solvent polarity by changing the extraction temperature. Relatively high pressure facilitates extraction above the boiling point of the solvent accelerating extraction process while lowering water usage and eliminating water losses due to evaporation (Dunford et al. 2010).

Therefore, the main objective of this study is to investigate cytotoxicity of pecan shell extracts obtained from two different varieties grown and commercially processed in Oklahoma on cancer cells. The impacts of the extraction temperature on chemical composition of the water extracts and their biological activity are also evaluated.

## Materials and methods

### Materials

#### Pecan shell samples

The shell samples were obtained directly from a pecan sheller operating in Oklahoma, USA. There were two different varieties, Pawnee grown in Sapulpa, OK, and Native from Bristow, OK, which were sampled and processed separately. The shelling process was described elsewhere (Sims 1994). The samples received in our laboratory were ground using a hammer mill first (Fitz Mill DAS06, Elmhurst, IL, USA) and then a 2nd time with a coffee grinder (Mr. Coffee W183ME, FL, USA). The ground samples were stored in plastic Ziploc bags at − 20 °C until further analysis.

#### Reagents

All the chemicals were analytical grade unless otherwise stated.

#### Cancer cell lines

The cancer cell lines A549 (human alveolar adenocarcinoma), HeLa (human cervix adenocarcinoma), MDA-MB-231 (human breast adenocarcinoma), PC-3 (human prostate adenocarcinoma), SK-MEL (human skin malignant melanoma), HT-29 (human colorectal adenocarcinoma) and Vero (Cercopithecus aethiops kidney epithelial cell) healthy cell line were from American Cell Culture Collection (ATCC, Manassas, VA).

### Methods

#### Proximate composition of pecan shells

Protein content of the samples was analyzed as nitrogen on a carbon–nitrogen analyzer (TruSpec CN, Leco USA, St. Joseph, MI) according to the method of Forage Analyses Procedures (Undersander et al. 1993).

Moisture, ash and lipid contents of the samples were determined according to the American Association of Cereal Chemists (AACC) method number 44 − 15A (AACC 2000), Association of Official Analytical Chemists (AOAC) method 923.03 and AOAC method 960.39, respectively (AOAC 2005).

#### Extraction

A detailed description of the accelerated solvent extraction system, ASE 350 (Thermo Scientific, Waltham, MA, USA), used for the extraction of pecan shells was reported earlier (Dunford et al. 2022; Dunford et al. 2010; Dunford and Zhang 2003). About 15 g pecan shell samples were filled into the 66 mL extraction cells. Extraction parameters, temperature, time, number of extraction steps, solvent composition, purge time and flush volume were maintained electronically according to the programmed set points: solvent 100% deionized water, 5 min static time, 3 cycles, 100% flush, 60 s purge at 1500 psi. Subcritical water extraction of the samples was carried out at four different temperatures, 80, 100, 125 and 150 °C. Nitrogen gas was used to purge the remaining extract into the collection bottle at the end of the extraction before unloading the cell. Then, solvent was evaporated from the extract/solvent mixtures at 40 °C under vacuum using a Rapidvap evaporator (Labconco, Kansas City, MO) until constant weight was attained. Wilmington, DE). The extracts were freeze-dried and stored at − 20 °C away from light until further analysis.

#### Chemical composition of shell extracts

Details of the HPLC method used for this study were published earlier elsewhere (Dunford et al. 2022). Chromatographic separation of the compounds present in pecan shell extracts was carried out using an HPLC system (1260 Infinity series, Agilent Technologies, Santa Clara, CA, USA) equipped with a pump, an online degasser, an auto sampler, and a Diode Array Detector (DAD). Elution of the peaks in the chromatogram was performed with a C18 reverse-phase column (4.6 mm × 25 cm), type Spherisorb ODS-2 5 μm, 100 A° (Waters Corporation, Milford, MA, USA) (Dunford et al. 2022). The detector wavelength was set at 280 nm. The column temperature was maintained at 35 °C. A ternary linear elution gradient consisting of water with 0.2% H_3_PO_4_ (v/v) (A), methanol (B) and acetonitrile (C) was used at a flow rate of 1.0 mL/min. Separation of the chemical species was achieved using the elution method shown in Table 1. Freeze-dried samples were dissolved in 80/20 methanol/water (v/v) solution for HPLC injection.

**Table 1:** HPLC mobile phase gradient

Time (min)	A (%)	B (%)	C (%)
0	96	2	2
40	50	25	25
45	40	30	30
50	0	50	50
52	0	50	50
55	96	2	2

#### Cytotoxic activity assay

The cells were cultured in DMEM Ham’s F12 or RPMI 1640 or supplemented with 10% fetal bovine serum, l–glutamine, (2 mmol/L), penicillin (100 U/mL), streptomycin (100 mg/mL). The standard MTT assay was used to determine the cytotoxic activity of the extracts. In summary, cells in exponential growth phase were placed in 96-well plates (6000 cells/well), and pecan shell extracts (1.5625–25 μg/mL DMSO) were added to each well. Then, the plates were incubated in a humidified atmosphere containing 5% CO_2_ at 37 °C for 48 h. Cell proliferation was determined by adding 0.5 mg/mL of Dulbecco’s phosphate-buffered saline (Gibco, USA) to each well. Medium was removed 4 h later, and the blue formazan crystals formed during the incubation were dissolved in 200 μL 100% dimethyl sulfoxate solution. Quantities of blue formazan product were measured at 570–690 nm by using a microplate reader (Versa-max, Tunable Microplate Reader, USA). The assays were carried out in three independent replicates. Cytotoxicity was determined according to the percent cell viability. There were three different controls, Positive control, Doxorubicin which is a commercial drug used in cancer treatment; negative control NC consists of cells + growth medium with no DMSO; and NC2 contains cells + growth medium + 0.1% DMSO. NC column in the data tables does not show the standard deviations, because they were less than 1%.

### Statistical analysis

All analytical tests for the sample characterization were carried out at least in duplicate. Cell viability tests were performed in triplicate. Means were compared using the least significance difference (Tukey’s test) method. All statistical tests were performed at the *p* = 0.05 level of significance.

Principal Component Analysis (PCA) and Spearman correlation coefficient calculations were performed using MINITAB 15 Statistical Software. Similarities and differences between main groups and observations were presented as score plots. The loading plots have been used to explain the relationship between variables in the score plots and cluster observations.

## Results and discussion

### Proximate composition

The nutshell samples from both varieties examined in this study, Native and Pawnee, contained higher amount of oil (Table 2) than expected (Sevimli-Gur et al. 2021). The latter results can be explained by the presence of nut meat in the shell samples. Efficiency of the mechanical nut meat and shell separation process used by the industry varies depending on the processing conditions and the pecan variety. Pecan halves or meat are reduced in size to small pieces during the shelling process (Sims 1994). Small meat pieces which are rich in oil content, over 65% by weight (Villarreal-Lozoya et al. 2007), end up in the byproducts and consequently increasing the oil content of the extracts obtained from the byproduct streams. Indeed, in a previous study carried out with the same pecan shell samples used in this study, we reported that fatty acids commonly found in pecan meat oil, oleic and linoleic acids, were among the major chemical compounds identified in the extracts obtained from the byproducts (Sevimli-Gur et al. 2021). It is also important to note the higher oil content of the byproduct streams from Native variety than that in Pawnee (Table 2). Physical structure and size of the nuts significantly affect the efficiency of shell and meat separation. Native variety produces smaller nuts than that of Pawnee cultivar, resulting in larger amount of meat remaining in the shells and higher oil content in the byproduct stream form Native variety.

**Table 2:** Proximate composition of shells from two different varieties (w/w, % ± SD)

Sample	Oil	Protein	Ash	Moisture
Pawnee (Sapulpa, OK)	1.3 ± 0.01	1.5 ± 0.01	2.0 ± 0.03	14.5 ± 0.3
Native (Bristow, OK)	2.8 ± 0.1	1.9 ± 0.1	1.7 ± 0.02	12.8 ± 0.03

### Phenolic composition of the extracts

**Fig. 1:**
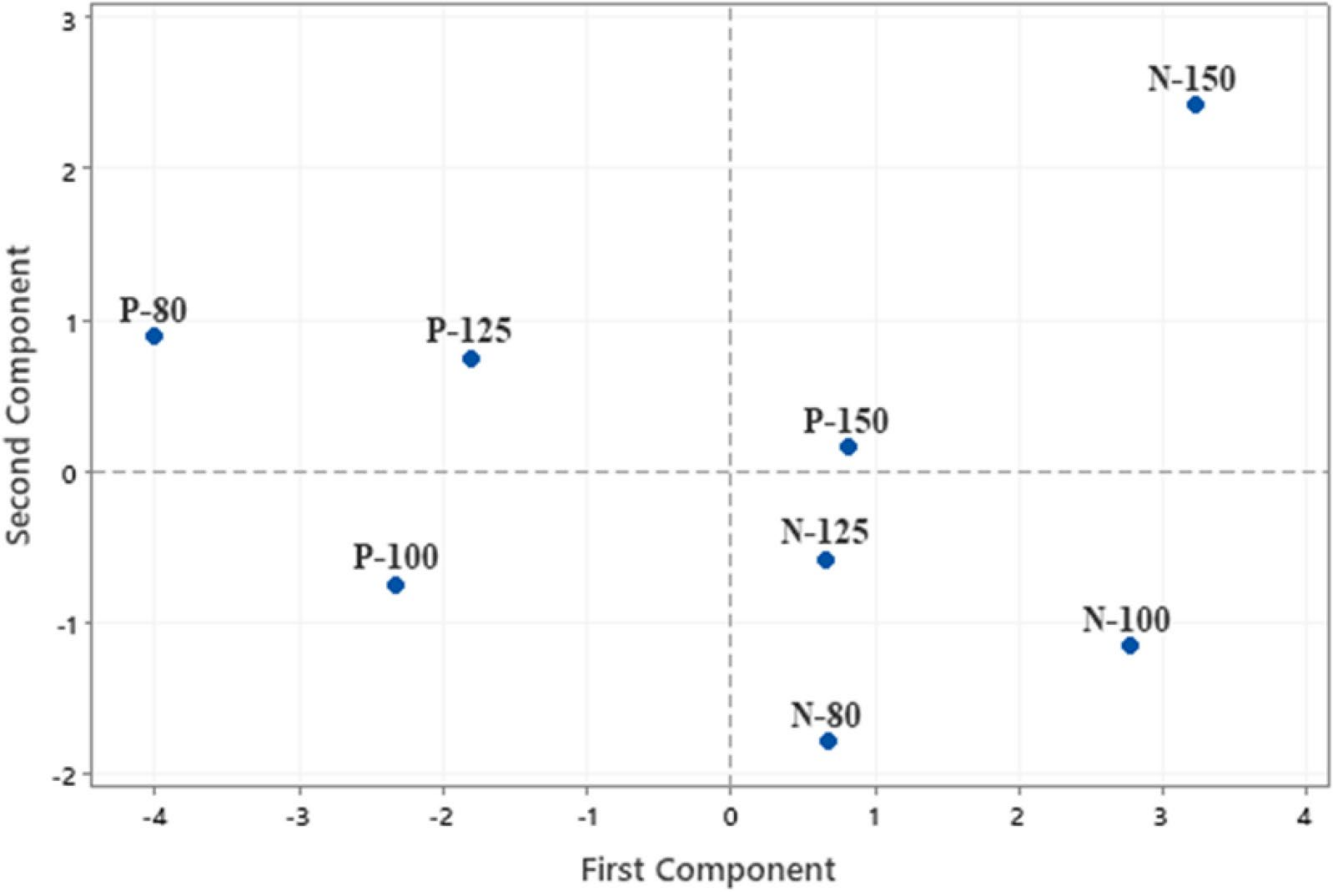
Principal Component Analysis (PCA) score plot for the pecan shell extracts

**Fig. 2:**
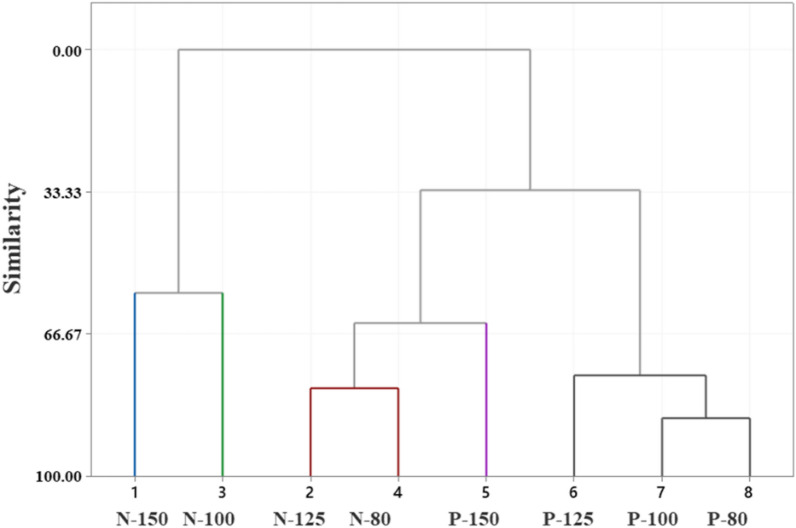
Dendrogram for the pecan shell extracts

**Fig. 3:**
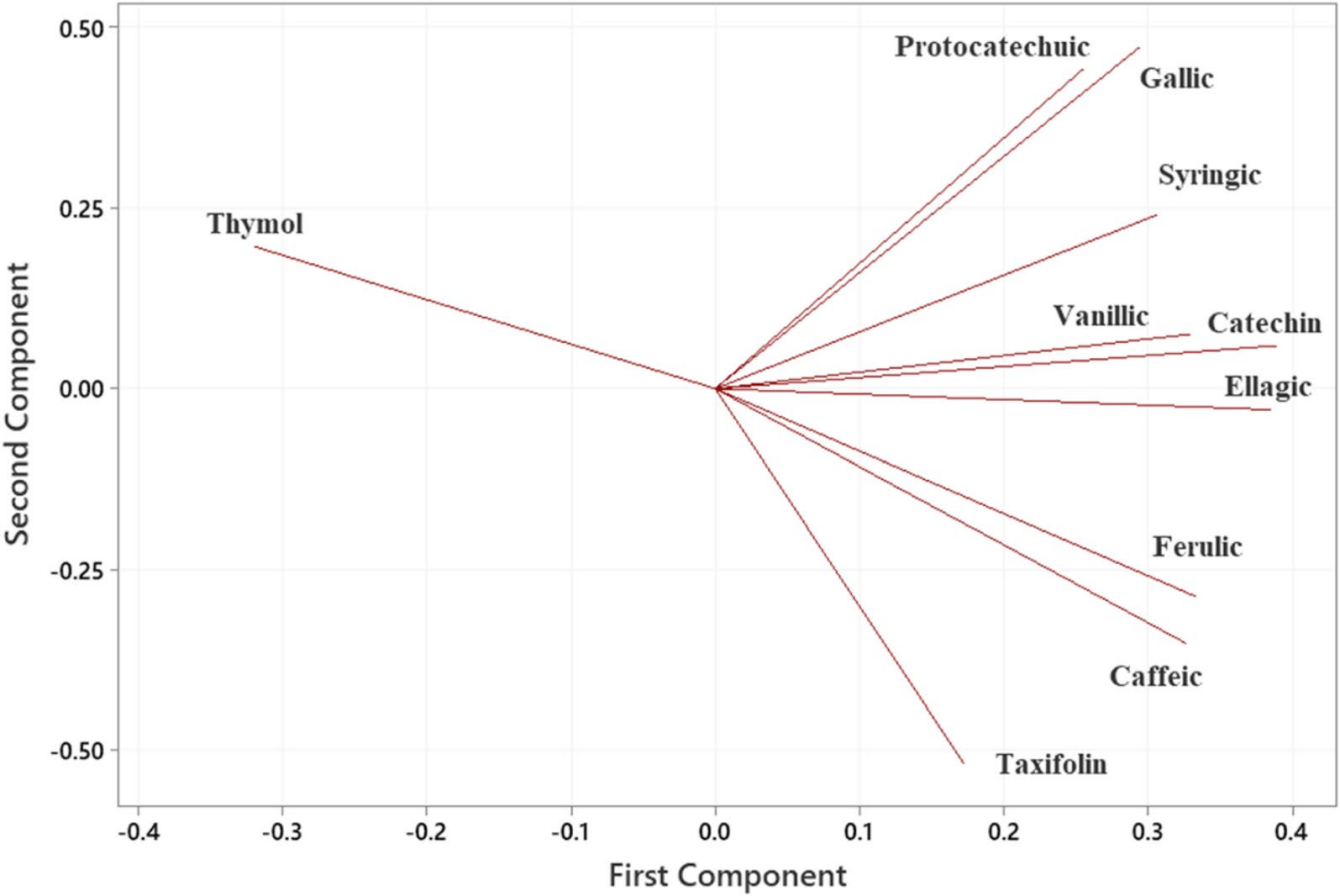
Principal Component Analysis (PCA) loading plot for the pecan shell extracts

Gallic, protocatechuic, catechin, caffeic and ellagic acids were the major phenolic compounds detected in all the extracts examined in this study (Table 3). Taxifolin, vanillic, syringic and thymol were the other phenolic compounds found in some extracts, but not all. The water extract obtained at 100 °C from the Native variety, N-100, had the highest concentration of ellagic acid, 59.7 µg/mL, among the samples analyzed. Considering that extract composition is significantly affected by several variables; extraction method, byproduct type and temperature, the compositional data were evaluated by a statistical analysis method known as Principal Component Analyses. Eigen analyses of the correlation matrix showed that first and second principal components account for 63.4 and 18.1% of the total variance, respectively. All the compounds identified in the samples except thymol were positively correlated with the first principal component. Ellagic acid (0.384) and catechin (0.388) were the compounds correlated the most with the first principal component. Gallic (0.472) and protocatechuic (0.442) correlated mostly with the second component. The score plot (Fig. 1) which displays the clusters, trends, and outliers in the first two principal components, revealed a broad scattering. The extract from Native variety obtained at 150 °C, N-150, was significantly away from the other samples on the plot. Mainly Gallic and protocatechuic acids were responsible for the separation of the extract N-150 from the others. Although the effect of the extraction temperature on total phenolic content of the extracts was not significant, *p* = 0.092, variety had a substantial effect on the total phenolic content of the samples (*p* = 0.017). The dendrogram (Fig. 2) further demonstrated the significant effect of variety on extract chemical composition. Considering that this study focuses on the characterization of the crude pecan shell extracts, the lack of a clear trend by extraction temperature is not surprising (Fig. 3).

**Table 3:** Phenolic composition of pecan shells from different varieties (µg/g)*

Sample ID/Phenolic compound	Gallic	Protocatechuic	Catechin	Vanillic	Caffeic	Syringic	Ferulic	Taxifolin	Ellagic	Thymol
P-150	25.0^b^ ± 0.1	16.2^b^ ± 0.5	20.9^b^ ± 0.6	n.d	4.3^b^ ± 0.2	n.d	9.1^c^ ± 0.1	3.9^d^ ± 0.1	42.0^c^ ± 5.4	n.d
P-125	15.5^d^ ± 0.4	9.9^c^ ± 1.6	10.3^d^ ± 0.2	n.d	1.6^d^ ± 0.1	3.0^d^ ± 0.1	2.8^f^ ± 0.1	1.2^ g^ ± 0.1	23.2^e^ ± 0.1	0.01^b^ ± 0.02
P-100	11.3^e^ ± 0.1	7.1^de^ ± 0.2	8.8^e^ ± 0.1	n.d	1.9^d^ ± 0.2	0.7^f^ ± 0.1	n.d	6.3^b^ ± 0.1	16.3^f^ ± 0.1	0.2^b^ ± 0.03
P-80	9.4^f^ ± 0.3	5.3^f^ ± 0.1	6.6^f^ ± 0.5	n.d	0.8^e^ ± 0.1	0.7^f^ ± 0.1	n.d	n.d	13.3^f^ ± 0.2	0.7^a^ ± 0.1
N-150	41.6^a^ ± 1.1	17.8^a^ ± 0.2	24.8^a^ ± 0.1	12.0^a^ ± 0.1	3.7^c^ ± 0.1	10.7^a^ ± 0.2	8.1^d^ ± 0.2	2.2^f^ ± 0.1	51.8^b^ ± 1.2	n.d
N-125	14.3^d^ ± 0.1	7.9^d^ ± 0.1	17.7^c^ ± 0.4	6.6^c^ ± 0.2	5.3^a^ ± 0.2	4.4^c^ ± 0.1	6.2^e^ ± 0.1	2.8^e^ ± 0.1	43.7^c^ ± 1.0	n.d
N-100	21.9^c^ ± 0.3	10.1^c^ ± 0.5	21.6^b^ ± 0.7	7.7^b^ ± 0.1	5.3^a^ ± 0.1	8.2^b^ ± 0.1	14.4^a^ ± 0.3	7.6^a^ ± 0.1	59.7^a^ ± 0.6	n.d
N-80	10.9^ef^ ± 0.1	6.1^ef^ ± 0.1	17.4^c^ ± 0.4	6.8^c^ ± 0.2	5.3^a^ ± 0.1	2.6^e^ ± 0.1	13.1^b^ ± 0.6	5.0^c^ ± 0.1	35.3^d^ ± 0.2	n.d

*The values with the same superscript letter in the same column are not significantly different at *p* = 0.05 level

Sample ID: Variety (N: Native, P: Pawnee)-Extraction temperature (°C)

**Fig. 4:**
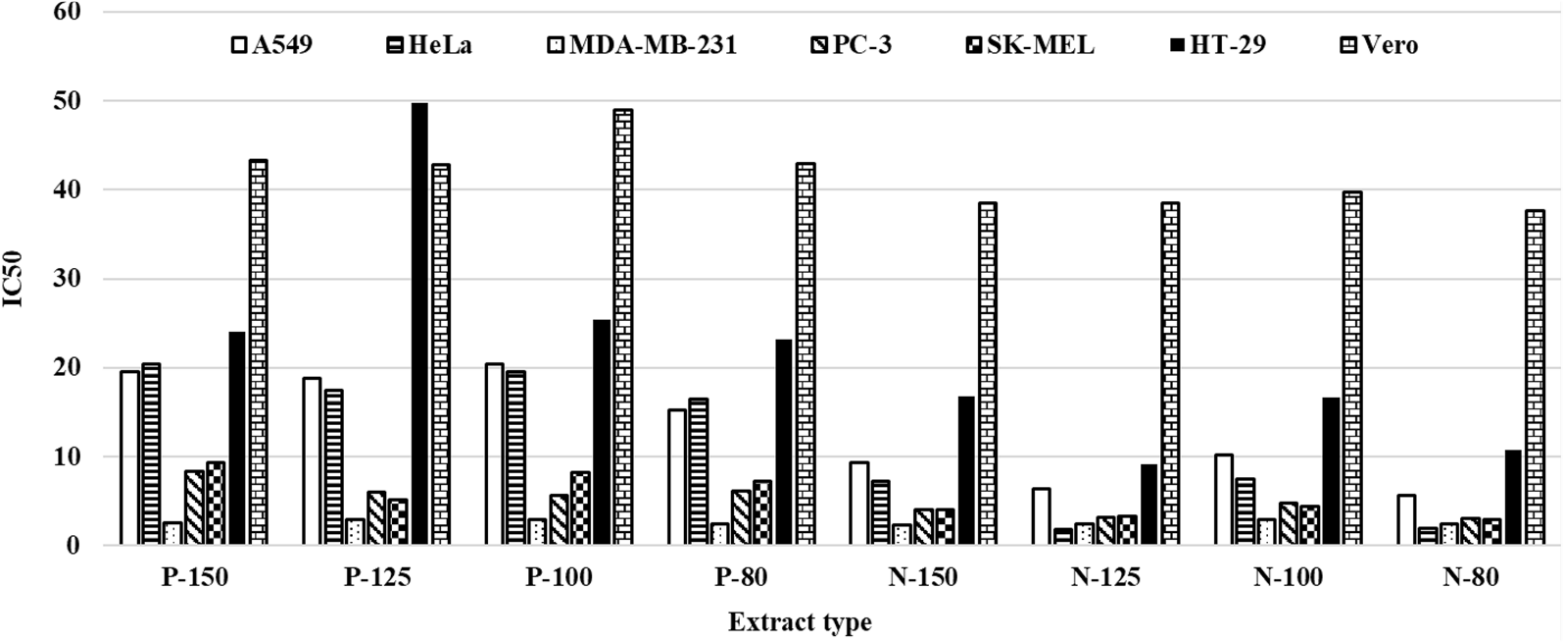
IC50 values as affected by type of the cell line, pecan variety and extract temperature*. *Sample ID: Variety (N: Native)-Extraction temperature (°C). The values with the same superscript letter in the same column are not significantly different at *p* = 0.05 level

### Cytotoxicity of the extracts on cancer cells

Doxorubicin which is a drug used in cancer chemotherapy was used as the positive control (PC) at 10 µg/mL concentration for the extracts. The negative controls (NC and NC2) did not contain any extract. In general, cell viability varied with the cell and extract type and the extract dose used (Tables 4 and 5). The highest cell proliferation, 346.6%, was observed with the cell line HT-29, human, colorectal adenocarcinoma cells, at the lowest extract, P-125, concentration examined, 1.5625 µL. This cell culture study also demonstrated that pecan shell extracts were not cytotoxic to healthy cells, Vero, meaning that cell viability did not decrease upon extract treatment within the concentration range investigated (Tables 4 and 5). As the concentration of the extract used for the cell treatment increased, cell viability decreased significantly for all the cell lines. PC, doxorubicin, was the most effective treatment with the A549 cells resulting in only 7.5% cell viability. ANOVA of the experimental data showed that variety, extraction temperature, extract type and concentration, cell line and their interactions had significant (*p* < 0.0001) effects on cell viability. The highest F value, 2726.46, was calculated for the cell line indicating that variation between cell lines was much higher than the variation within a cell line. Analyses of the IC50 data (Fig. 4) verified a similar trend, the effects of pecan variety, extraction temperature, cell type and their interactions on IC50 were significant (*p* < 0.0001) and the highest F value, 532.02, was for the cell line followed by nut variety, F = 307.55.

**Table 4:** Effect of cell line, extraction temperature and concentration of the extracts obtained from Pawnee variety on cell viability (%)* as compared to controls

Sample ID	Cell line	NC	NC2	PC	Extract concentration (µg/mL)				
					25	12.5	6.25	3.125	1.5625
P-150	A549	137.3 ± 13.6	100.0	7.4 ± 0.6	26.8 ± 4.4	87.7 ± 12.4	192.9 ± 26.4	211.0 ± 28.8	229.6 ± 31.3
	HeLa	101.9 ± 10.0	100.0	32.9 ± 3.1	34.9 ± 5.5	87.5 ± 12.4	105.2 ± 14.8	165.5 ± 22.8	181.5 ± 24.9
	MDA-MB-231	127.9 ± 12.6	100.0	14.3 ± 1.3	3.3 ± 0.4	5.5 ± 0.7	3.9 ± 1.3	44.9 ± 6.8	117.4 ± 16.4
	PC-3	120.5 ± 11.9	100.0	46.8 ± 4.5	4.8 ± 1.5	6.0 ± 1.6	58.2 ± 8.5	145.7 ± 20.1	213.3 ± 29.1
	SK-MEL	192.3 ± 19.1	100.0	28.3 ± 2.7	7.2 ± 0.5	19.0 ± 3.3	68.6 ± 9.9	135.3 ± 18.8	187.3 ± 25.7
	HT-29	177.2 ± 17.6	100.0	38.2 ± 3.7	49.3 ± 7.4	125.2 ± 17.4	160.1 ± 22.1	205.0 ± 28.0	327.4 ± 44.2
	Vero	168.9 ± 16.7	100.0	125.2 ± 12.4	178.0 ± 25.0	190.3 ± 26.7	221.6 ± 31.1	239.5 ± 33.5	317.3 ± 44.3
P-125	A549	137.3 ± 13.6	100.0	7.4 ± 0.6	28.4 ± 2.3	92.8 ± 6.4	204.3 ± 13.6	223.4 ± 14.8	243.1 ± 16.1
	HeLa	101.9 ± 10.0	100.0	32.9 ± 3.1	37.0 ± 2.8	92.6 ± 6.4	111.4 ± 7.6	175.2 ± 11.7	192.1 ± 12.8
	MDA-MB-231	127.9 ± 12.6	100.0	14.3 ± 1.3	3.5 ± 0.2	5.8 ± 0.4	4.1 ± 0.7	47.6 ± 3.5	124.3 ± 8.4
	PC-3	120.5 ± 11.9	100.0	46.8 ± 4.5	5.1 ± 0.8	6.4 ± 0.8	61.7 ± 4.4	154.2 ± 10.4	225.9 ± 15.0
	SK-MEL	192.3 ± 19.1	100.0	28.3 ± 2.7	7.6 ± 0.2	20.1 ± 1.7	72.6 ± 5.1	143.3 ± 9.7	198.3 ± 13.2
	HT-29	177.2 ± 17.6	100.0	38.2 ± 3.7	52.2 ± 3.8	132.6 ± 9.0	169.5 ± 11.3	217.1 ± 14.4	346.6 ± 22.7
	Vero	168.9 ± 16.7	100.0	125.2 ± 12.4	188.4 ± 12.9	201.5 ± 13.7	234.6 ± 16.0	253.5 ± 17.2	335.9 ± 22.8
P-100	A549	137.3 ± 13.6	100.0	7.4 ± 0.6	27.7 ± 2.2	90.6 ± 6.3	199.3 ± 13.3	218.0 ± 14.5	237.2 ± 15.8
	HeLa	101.9 ± 10.0	100.0	32.9 ± 3.1	36.1 ± 2.8	90.4 ± 6.3	108.7 ± 7.5	170.9 ± 11.5	187.5 ± 12.6
	MDA-MB-231	127.9 ± 12.6	100.0	14.3 ± 1.3	3.4 ± 0.2	5.7 ± 0.4	4.0 ± 0.7	46.4 ± 3.4	121.3 ± 8.3
	PC-3	120.5 ± 11.9	100.0	46.8 ± 4.5	5.0 ± 0.7	6.2 ± 0.8	60.2 ± 4.3	150.5 ± 10.2	220.4 ± 14.7
	SK-MEL	192.3 ± 19.1	100.0	28.3 ± 2.7	7.5 ± 0.2	19.6 ± 1.7	70.8 ± 5.0	139.8 ± 9.5	193.5 ± 13.0
	HT-29	177.2 ± 17.6	100.0	38.2 ± 3.7	50.9 ± 3.7	129.4 ± 8.8	165.4 ± 11.1	211.8 ± 14.1	338.2 ± 22.3
	Vero	168.9 ± 16.7	100.0	125.2 ± 12.4	183.9 ± 12.6	196.6 ± 13.5	229.0 ± 15.7	247.4 ± 16.9	327.8 ± 22.4
P-80	A549	137.3 ± 13.6	100.0	7.4 ± 0.6	22.8 ± 2.5	74.6 ± 7.2	164.2 ± 15.2	179.6 ± 16.6	195.4 ± 18.1
	HeLa	101.9 ± 10.0	100.0	32.9 ± 3.1	29.7 ± 3.2	74.5 ± 7.2	89.5 ± 8.5	140.8 ± 13.1	154.5 ± 14.4
	MDA-MB-231	127.9 ± 12.6	100.0	14.3 ± 1.3	2.8 ± 0.2	4.7 ± 0.4	3.3 ± 0.8	38.3 ± 3.9	100.0 ± 9.5
	PC-3	120.5 ± 11.9	100.0	46.8 ± 4.5	4.1 ± 0.8	5.1 ± 0.9	49.6 ± 4.9	124.0 ± 11.6	181.6 ± 16.8
	SK-MEL	192.3 ± 19.1	100.0	28.3 ± 2.7	6.1 ± 0.3	16.1 ± 1.9	58.4 ± 5.7	115.2 ± 10.8	159.4 ± 14.8
	HT-29	177.2 ± 17.6	100.0	38.2 ± 3.7	42.0 ± 4.3	106.6 ± 10.1	136.3 ± 12.7	174.5 ± 16.2	278.7 ± 25.5
	Vero	168.9 ± 16.7	100.0	125.2 ± 12.4	151.5 ± 14.4	162.0 ± 15.4	188.6 ± 17.9	203.8 ± 19.4	270.1 ± 25.6

*Sample ID: Variety (P: Pawnee)-Extraction temperature (°C)

**Table 5:** Effect of cell line, extraction temperature and concentration of the extracts obtained from Native variety on cell viability (%)* as compared to controls

Sample ID	Cell line	NC	NC2	PC	Extract concentration (µg/mL)				
					25	12.5	6.25	3.125	1.5625
N-150	A549	137.3 ± 13.6	100.0	7.4 ± 0.6	17.5 ± 1.5	45.8 ± 4.3	70.9 ± 6.7	73.4 ± 7.1	76.1 ± 7.4
	HeLa	101.9 ± 10.0	100.0	32.9 ± 3.1	21.3 ± 1.9	45.7 ± 4.3	53.9 ± 5.2	59.3 ± 5.7	59.6 ± 5.7
	MDA-MB-231	127.9 ± 12.6	100.0	14.3 ± 1.3	1.9 ± 0.05	4.8 ± 0.3	6.8 ± 0.5	25.9 ± 2.4	59.6 ± 5.7
	PC-3	120.5 ± 11.9	100.0	46.8 ± 4.5	7.3 ± 0.5	7.8 ± 0.6	32.1 ± 3.0	66.8 ± 6.5	68.5 ± 6.6
	SK-MEL	192.3 ± 19.1	100.0	28.3 ± 2.7	1.7 ± 0.06	13.9 ± 1.2	36.9 ± 3.5	68.0 ± 6.6	92.1 ± 9.0
	HT-29	177.2 ± 17.6	100.0	38.2 ± 3.7	28.0 ± 2.6	63.2 ± 6.1	79.5 ± 7.7	100.4 ± 9.8	103.7 ± 10.1
	Vero	168.9 ± 16.7	100.0	125.2 ± 12.4	146.7 ± 14.4	152.6 ± 15.0	167.3 ± 16.5	180.2 ± 17.8	185.3 ± 18.3
N-125	A549	137.3 ± 13.6	100.0	7.4 ± 0.6	13.1 ± 1.1	34.4 ± 3.2	53.2 ± 5.1	55.1 ± 5.2	57.1 ± 5.4
	HeLa	101.9 ± 10.0	100.0	32.9 ± 3.1	16.0 ± 1.3	34.3 ± 3.2	40.5 ± 3.8	44.5 ± 4.2	54.8 ± 5.2
	MDA-MB-231	127.9 ± 12.6	100.0	14.3 ± 1.3	1.4 ± 0.1	3.6 ± 0.09	5.1 ± 0.3	19.5 ± 1.7	64.8 ± 6.2
	PC-3	120.5 ± 11.9	100.0	46.8 ± 4.5	5.5 ± 0.3	5.9 ± 0.3	24.1 ± 2.1	50.1 ± 4.8	51.4 ± 4.9
	SK-MEL	192.3 ± 19.1	100.0	28.3 ± 2.7	1.3 ± 0.1	10.4 ± 0.8	27.7 ± 2.5	51.0 ± 4.8	69.1 ± 6.7
	HT-29	177.2 ± 17.6	100.0	38.2 ± 3.7	21.0 ± 1.8	47.5 ± 4.5	59.6 ± 5.7	75.3 ± 7.3	77.8 ± 7.5
	Vero	168.9 ± 16.7	100.0	125.2 ± 12.4	110.1 ± 10.7	114.6 ± 11.2	125.6 ± 12.3	135.3 ± 13.3	139.1 ± 13.6
N-100	A549	137.3 ± 13.6	100.0	7.4 ± 0.6	17.2 ± 1.4	45.0 ± 4.2	69.7 ± 6.6	72.1 ± 7.1	74.8 ± 7.1
	HeLa	101.9 ± 10.0	100.0	32.9 ± 3.1	20.9 ± 1.7	45.0 ± 4.1	53.0 ± 4.9	58.3 ± 5.5	71.7 ± 6.8
	MDA-MB-231	127.9 ± 12.6	100.0	14.3 ± 1.3	1.8 ± 0.2	4.7 ± 0.1	6.7 ± 0.3	25.5 ± 2.2	84.8 ± 8.1
	PC-3	120.5 ± 11.9	100.0	46.8 ± 4.5	7.1 ± 0.4	7.7 ± 0.4	31.6 ± 2.8	65.7 ± 6.2	67.3 ± 6.4
	SK-MEL	192.3 ± 19.1	100.0	28.3 ± 2.7	1.6 ± 0.2	13.6 ± 1.0	36.3 ± 3.3	66.8 ± 6.3	90.6 ± 8.7
	HT-29	177.2 ± 17.6	100.0	38.2 ± 3.7	27.5 ± 2.4	62.2 ± 5.9	78.1 ± 7.5	98.7 ± 9.5	101.9 ± 9.8
	Vero	168.9 ± 16.7	100.0	125.2 ± 12.4	144.2 ± 14.1	150.1 ± 14.6	164.5 ± 16.1	177.2 ± 17.4	182.2 ± 17.9
N-80	A549	137.3 ± 13.6	100.0	7.4 ± 0.6	13.1 ± 1.2	34.3 ± 3.3	53.1 ± 5.2	54.9 ± 5.4	56.9 ± 5.6
	HeLa	101.9 ± 10.0	100.0	32.9 ± 3.1	15.9 ± 1.5	34.2 ± 3.3	40.4 ± 3.9	44.4 ± 4.3	54.6 ± 5.3
	MDA-MB-231	127.9 ± 12.6	100.0	14.3 ± 1.3	1.4 ± 0.01	3.6 ± 0.2	5.1 ± 0.4	19.4 ± 1.8	64.6 ± 6.3
	PC-3	120.5 ± 11.9	100.0	46.8 ± 4.5	5.4 ± 0.4	5.9 ± 0.5	24.0 ± 2.3	50.0 ± 4.9	51.3 ± 5.0
	SK-MEL	192.3 ± 19.1	100.0	28.3 ± 2.7	1.3 ± 0.003	10.4 ± 0.9	27.6 ± 2.6	50.9 ± 5.0	69.0 ± 6.8
	HT-29	177.2 ± 17.6	100.0	38.2 ± 3.7	20.9 ± 2.0	47.4 ± 4.6	59.5 ± 5.8	75.2 ± 7.4	77.6 ± 7.6
	Vero	168.9 ± 16.7	100.0	125.2 ± 12.4	109.8 ± 10.9	114.3 ± 11.3	125.3 ± 12.4	134.9 ± 13.4	138.7 ± 13.8

*Sample ID: Variety (N: Native)-Extraction temperature (°C)

Some of the pecan shell extracts were significantly more effective than the PC in reducing cell viability. For example, all the extracts from Pawnee variety were very effective on the cell line MDA-MB-231 (human breast adenocarcinoma), with significantly lower cell viability, 3.3–4.1%, at a lower extract concentration 6.25 µg/mL than that of the PC, 7.4%, at 10 µg/mL concentration which corresponds to over 40% higher efficiency of the extracts on MDA-MB-231. The extracts from the Native variety had a similar trend on the same cell line, MDA-MB-231, and concentration (6.25 µg/mL), with slightly higher cell viability, 5.1–6.8% than the extracts from Pawnee variety. The lowest IC50 values, 1.8–1.9 µg/mL were obtained with the extracts N-125 and N-80 on the cell line HeLa (human cervix adenocarcinoma). The Spearman correlation coefficient between total and individual phenolic content of the extracts and IC50 values were negative, except thymol, Gallic and protocatechuic acid contents. The correlations between caffeic (− 0.294, *p* = 0.028) and vanillic (− 0.281, *p* = 0.036) acid contents of the extracts and the IC50 values were significant at *p* = 0.05 level. The low correlation coefficients between individual phenolic compounds and IC50 values can be explained by the very complex chemical composition of the crude extracts.

## Conclusions

To the best of our knowledge, this is the first study reporting the effects of subcritical water extracts obtained from the byproducts generated at commercial pecan shelling facilities on the cell lines representing a broad range of cancer types. The human cervical, breast, prostate, colorectal, skin and lung cancer cells were treated with pecan shell water extracts. Two different pecan varieties were extracted at four different temperatures between 80 and 150 °C. This study also examined the correlations between the total and individual phenolic contents of the extracts, cell viability and IC50 values. The experimental results established that pecan shell extracts did not have cytotoxic effect on the healthy cells. Although the extraction temperature did not substantially affect the phenolic content of the extracts, pecan variety had a significant effect on the extract composition. The highest cell proliferation, 346.6%, was measured with the cell line HT-29, human, colorectal adenocarcinoma cells, at the lowest extract, P-125, concentration examined, 1.5625 µL. The effects of cell line, extraction temperature, extract concentration, pecan variety and their interactions on the cell viability and the corresponding IC50 were all significant. Even at a lower dosage, some of the pecan shell extracts were significantly more effective, over 40%, than Doxorubicin, a drug used in cancer chemotherapy, in reducing the cell viability, specifically on human breast cancer cells. Significant negative correlations were found between caffeic and vanillic acid contents of the extracts and the IC50 values calculated for the cell lines examined in this study.

The findings of this study clearly demonstrated the beneficial effects of pecan shell extracts on various human cancer cells, indicating the valorization potential of the byproducts generated at commercial pecan shelling operations. There is no doubt that any effort toward the development of alternative cancer treatment tools to the current synthetic drugs is a meritorious endeavor. Although the data presented in this article expand our understanding of the interactions between plant extracts and the cancer cells, further research is needed to decipher the biological pathways involved in the process. Considering that the samples used in this study were crude extracts with very complex chemical composition, it is expected that downstream processing and purification of the extracts will further enhance their biological activity and facilitate development of formulations for specific applications. The practical implications of the data generated in this study are noteworthy because they would support and encourage further research in the field.

## Data Availability

All data generated or analysed during this study are included in this published article.

## References

[CR1] AACC (2000) American Association Of Cereal Chemists. Approved Methods. Aacc, St. Paul, Mn

[CR2] AOAC (2005) Official Methods Of Analysis. 18th Edn. Association Of Official Analytical Chemists, Wahington, D.C., Usa

[CR3] Atanasov AG, Sabharanjak SM, Zengin G (2018). Pecan nuts: a review of reported bioactivities and health effects. Trends Food Sci Technol.

[CR4] Baek SJ, Kim J-S, Jackson FR, Eling TE, Mcentee MF, Lee S-H (2004). Epicatechin Gallate-induced expression of Nag-1 is associated with growth inhibition and apoptosis in colon cancer cells. Carcinogenesis.

[CR5] Cason C, Vk Y, Moreira J, Adhikari A (2021). Antioxidant properties of pecan shell bioactive components of different cultivars and extraction methods. Foods.

[CR6] Cogan B, Rc P, Cm P, Nt J, Ja C (2023). Pecan-enriched diet improves cholesterol profiles and enhances postprandial microvascular reactivity in older adults. Nutr Res.

[CR7] Do Prado ACP, Aragão AM, Fettand R, Block JM (2009). Antioxidant properties of pecan nut [Carya Illinoinensis (Wangenh.) C. Koch] shell infusion. Grasas Aceites.

[CR8] Do Prado ACP, Manion BA, Seetharaman K, Deschamps FC, Barrera Arellano D, Block JM (2013). Relationship between antioxidant properties and chemical composition of the oil and the shell of pecan nuts [Caryaillinoinensis (Wangenh.) C. Koch]. Ind Crops Prod.

[CR9] Do Prado ACP, Da Silva HS, Da Silveira SM (2014). Effect of the extraction process on the phenolic compounds profile and the antioxidant and antimicrobial activity of extracts of pecan nut [Carya Illinoinensis (Wangenh.) C. Koch] shell. Ind Crops Prod.

[CR10] Dunford NT, Zhang M (2003). Pressurized solvent extraction of wheat germ oil. Food Res Int.

[CR11] Dunford NT, Irmak S, Jonnala RS (2010). Pressurized solvent extraction of policosanol from wheat straw, germ and bran. Food Chem.

[CR12] Dunford NT, Gumus ZP, Gur CS (2022). Chemical composition and antioxidant properties of pecan shell water extracts. Antioxidants.

[CR13] FDA (2003) Qualified Health Claims: Letter Of Enforcement Discretion-Nuts And Coronory Heart Disease. U.S. Food And Drug Administration. In. http://www.Cfsan.Fda.Gov/~Dms/Qhcnuts2.html Accessed November 6, 2010

[CR14] FDA (2015) Dietary Guidelines For Americans 2015–2020. In: (Fda) Ufada (Ed). 8th Edn

[CR15] Guarneiri LL, Paton CM, Cooper JA (2021). Pecan-enriched diets alter cholesterol profiles and triglycerides in adults at risk for cardiovascular disease in a randomized, controlled trial. J Nutr.

[CR16] Hilbig J, Pdb P, Vmads G (2018). Aqueous extract from pecan nut [*Carya Illinoinensis* (Wangenh) C. Koch] shell show activity against breast cancer cell line Mcf-7 and ehrlich ascites tumor In Balb-C mice. J Ethnopharmacol.

[CR17] Porto LCS, Silva JD, Sousa K (2016). Evaluation of toxicological effects of an aqueous extract of shells from the pecan nut Carya Illinoinensis (Wangenh.) K. Koch and the possible association with its inorganic constituents and major phenolic compounds. Evid-Based Complement Alternat Med.

[CR18] Prado ACP, Aragão AM, Fett R, Block JM (2009). Phenolic compounds and antioxidant activity of pecan [Carya illinoinensis (Wangenh.) C. Koch] Shell Extracts. Braz J Food Technol.

[CR19] Sevimli-Gur C, Gezgin Y, Oz A, Sa S, Zp G, Nt D (2021). Biological activity of the extracts from pecan shelling industry byproducts. Trans ASABE.

[CR20] Sims KA (1994) Mechanization Of Post-Harvest Pecan Processing. In: Santerre Cr (Ed) Pecan Technology. Springer Us, Boston, Ma, P 68–86

[CR21] Tong X, Szacilo A, Chen H, Tan L, Kong L (2022). Using rich media to promote knowledge on nutrition and health benefits of pecans among young consumers. J Agric Food Res.

[CR22] Trevizol F, Dm B, Rcs B (2011). Comparative study between two animal models of extrapyramidal movement disorders: prevention and reversion by pecan nut shell aqueous extract. Behav Brain Res.

[CR23] Undersander D, Mertens Dr, Thiex N (1993) Forage Analyses Procedures. National Forage Testing Association, Omaha, Ne

[CR24] Villarreal-Lozoya JE, Lombardini L, Cisneros-Zevallos L (2007). Phytochemical constituents and antioxidant capacity of different pecan [Carya Illinoinensis (Wangenh.) K. Koch] cultivars. Food Chem.

